# Sensitivity and specificity of measuring children's free-living cycling with a thigh-worn Fibion® accelerometer

**DOI:** 10.3389/fspor.2023.1113687

**Published:** 2023-05-23

**Authors:** Arto J. Pesola, Samad Esmaeilzadeh, Pirjo Hakala, Nina Kallio, Päivi Berg, Marko Havu, Tiina Rinne

**Affiliations:** ^1^ Active Life Lab, South-Eastern Finland University of Applied Sciences, Mikkeli, Finland; ^2^ Juvenia – Youth Research and Development Centre, South-Eastern Finland University of Applied Sciences, Mikkeli, Finland; ^3^ Department of Neuroscience and Biomedical Engineering, Aalto University, Espoo, Finland; ^4^ Department of Built Environment, Aalto University, Espoo, Finland

**Keywords:** cycling, walking, active travel, accelerometer, energy expenditure, METS (metabolic equivalent of tasks), moderate-to-vigorous activity

## Abstract

**Objective:**

Cycling is an important part of children's active travel, but its measurement using accelerometry is a challenge. The aim of the present study was to evaluate physical activity duration and intensity, and sensitivity and specificity of free-living cycling measured with a thigh-worn accelerometer.

**Methods:**

Participants were 160 children (44 boys) aged 11.5 ± 0.9 years who wore a triaxial Fibion® accelerometer on right thigh for 8 days, 24 h per day, and reported start time and duration of all cycling, walking and car trips to a travel log. Linear mixed effects models were used to predict and compare Fibion-measured activity and moderate-to-vigorous activity duration, cycling duration and metabolic equivalents (METs) between the travel types. Sensitivity and specificity of cycling bouts during cycling trips as compared to walking and car trips was also evaluated.

**Results:**

Children reported a total of 1,049 cycling trips (mean 7.08 ± 4.58 trips per child), 379 walking trips (3.08 ± 2.81) and 716 car trips (4.79 ± 3.96). There was no difference in activity and moderate-to-vigorous activity duration (*p* > .105), a lower cycling duration (−1.83 min, *p* < .001), and a higher MET-level (0.95, *p* < .001) during walking trips as compared to cycling trips. Both activity (−4.54 min, *p* < .001), moderate-to-vigorous activity (−3.60 min, *p* < .001), cycling duration (−1.74 min, *p* < .001) and MET-level (−0.99, *p* < .001) were lower during car trips as compared to cycling trips. Fibion showed the sensitivity of 72.2% and specificity of 81.9% for measuring cycling activity type during the reported cycling trips as compared to walking and car trips when the minimum required duration for cycling was less than 29 s.

**Conclusions:**

The thigh-worn Fibion® accelerometer measured a greater duration of cycling, a lower MET-level, and a similar duration of total activity and moderate-to-vigorous activity during free-living cycling trips as compared to walking trips, suggesting it can be used to measure free-living cycling activity and moderate-to-vigorous activity duration in 10–12-year-old children.

## 1. Introduction

Children's daily physical activity accumulates from several sources, including school time, active hobbies, free play, and active travel. From these, active travel can form 20%–40% of daily moderate-to-vigorous physical activity, given it is a frequent, habitual part of daily living (1, 2). The most common forms of active travel are walking and cycling, with cycling being particularly common in Northern Europe and Scandinavian countries (3).

Cycling is more consistently associated with health outcomes, such as lower adiposity and cholesterol concentration, and a higher physical fitness, when compared to walking in children and adolescents (4–8). One possible reason is that habitual cycling is more intense as compared to walking (9). Measuring free-living cycling, and physical activity level during cycling, is important to better emphasize these benefits, and to promote cycling as part of children's physical activity habits and public health.

Device-based methods, primarily accelerometers, have improved free-living physical activity and moderate-to-vigorous physical activity measurement accuracy, but measuring free-living cycling remains a challenge. This is mainly because device impacts during cycling at typical wear locations like leg, trunk, or wrist, are minimal and not in proportion to the energy expenditure of cycling (10–13). Accordingly, children's free-living cycling energy expenditure and moderate-to-vigorous activity is under-estimated when using methodology assuming a linear association between accelerometer-counts and energy expenditure (14, 15). Validity is improved considerably when cycling is measured with multiple accelerometers or an accelerometer accompanied with other sensor, like heart rate, temperature, pressure, or GPS sensor (16–20). However, using these methods in larger samples and during free-living can be burdensome for the researchers and participants. Another approach for a single accelerometer system is to first recognize physical activity type (including, e.g., sitting, standing, walking and cycling), and then estimate energy expenditure for each of these activity types (21, 22). Since this method does not assume a linear association between accelerometer output and energy expenditure, the cycling energy expenditure estimate is possibly more valid as compared to estimates from more traditional methods.

Most of the studies validating different devices and analytical decisions in relation to cycling intensity have been performed in controlled laboratory conditions, where laboratory-grade criterion measures can be used. Given children's free-living activities are often sporadic and omnidirectional, the methods used to estimate their activities should be validated in free-living conditions (23). Given the mentioned challenges in measuring free-living cycling, proper criterion measures are also less available. Active travel habits are often measured with questionnaires or travel logs, which provide excellent individual-level validity for travel mode and are easier to use in larger samples as compared to device-based measures, like GPS sensors (24). Therefore, comparing device-measured physical activity during questionnaire-based travel modes can provide a possibility to evaluate the relative physical activity level between these travel modes in free-living conditions.

The first aim of this study was to quantify cycling duration, moderate-to-vigorous physical activity duration and metabolic equivalent of task (MET) level measured with a thigh-worn Fibion® accelerometer during free-living cycling trips, and to compare these to free-living walking (active reference) and car (sedentary reference) trips in 10–12-year-old children. The second aim was to study the sensitivity and specificity of Fibion® in measuring cycling during the cycling trips as compared to the walking and car trips.

## 2. Methods

Data was collected in FREERIDE project from 10 to 12-year-old children living in two South-Eastern Finnish cities, Mikkeli and Kouvola (25, 26). After receiving permission to recruit participants through schools, children and their parents were contacted through 11 primary schools in Mikkeli and 10 primary schools in Kouvola. Study info sheets and informed consent forms were delivered to teachers and taken to homes via children. The participants for the present study were a total of 160 children who returned informed consent signed by their parents and were willing to participate based on oral consent from themselves (*n* = 461), provided accelerometer and travel log data from same days (*n* = 365), and finally, reported any cycling, walking or car trip and had accelerometer data from these time segments (*n* = 160). Study protocol was approved by Aalto University Research Ethics Committee on 10th October 2019 and data collection was done in Spring 2021 during snow-free time.

Questionnaire data was collected at schools during one lesson totaling approximately 45 min, and accelerometer and travel log data was collected during the following 8 days. At school, the researcher first demonstrated how to complete the questionnaires, and children filled in the questionnaire on internet browser, while the researcher was available for questions and assistance. Subsequently, the researcher assisted in wearing the accelerometers, delivered extra medical adhesives and travel logs, and gave instructions for these measurements. This was done for one child at a time, either during recess or the next lesson.

### 2.1. Measurements

*Questionnaire* included questions about background variables and visits to different destinations and travel modes, which are reported elsewhere (25, 26).

*Travel log* was developed based on Helsinki region travel survey and was tailored for the purposes of the present study (27). Children were asked to report the exact start time of each trip, trip destination (school, home, organized sports, other organized activity, play, friends, shopping, or other), the primary travel mode (walk, bike, car, bus or school taxi, walking or cycling to transit stop, skate or scoot, other), as well as duration of the trip (categorical variable: less than 5, 5–15, 16–30, 31–45 min, and more than 45 min). For the purposes of accelerometer measurement, the children were also asked to report their waking up time, time when going to sleep, any non-wear periods, as well as whether the day was not a typical day (e.g., because being sick). A sample travel log is presented in Supplementary Material.

*Accelerometer* Children were wearing a Fibion® device (20 g, *L* = 30 mm, *W* = 32 mm, *T* = 10 mm; Fibion Inc, Jyväskylä, Finland) for 8 days, 24 h per day. The device was attached on the centerline of the anterior side of the thigh, one third from the proximal end, and secured in a waterproof covering with medical adhesive tape. The Fibion® device measures raw acceleration on three axes with an internal sampling rate of 12.5 Hz. The Fibion® device has no buttons or display and can operate for around 30 days on full charge condition. Fibion® is valid in estimating moderate-to-vigorous physical activity and energy expenditure against indirect calorimetry, which were the variables used in the present study (21, 22) Moreover, Fibion® has an overall accuracy of 85%–89% in detecting different activity types, with high accuracy (94%–100%) for detecting prone and supine lying, sitting, and standing. Fibion® has good to excellent validity for measuring sedentary (sitting) and upright (standing and walking) time against the ActivPAL4 monitor (22). Similar to ActivPAL4, Fibion has poorer reliability for measuring free-living cycling duration (28). However, moderate-to-vigorous activity during cycling or sensitivity and specificity in measuring children's free-living cycling has not been investigated.

### 2.2. Outcomes

*Cycling, walking and car trips* were segmented from Fibion data based on start time and duration of each trip reported in the Travel log (Supplementary Material). These trip types were selected because they are common in children's everyday life. Walking serves as a reference trip type for cycling because walking is typically more accurately detected with thigh-worn accelerometers than cycling (28). Car serves as a passive reference for cycling, since car trips should be less active than any active trips, including cycling trips. Travel log may be prone to recall error and given the trip duration was asked as a categorical variable, the trip duration segmented based on the travel log likely includes also other activities than the active travel. However, we assume these errors are similar between travel modes, and therefore by estimating differences between these travel modes should effectively eliminate such errors.

*Activity duration, Moderate-to-vigorous activity duration* and *Cycling duration* were calculated as a sum over each trip segment.

*Metabolic equivalent of tasks (METs)* were calculated by dividing the Fibion-estimated total energy expenditure of each trip segment by resting energy expenditure estimated individually for each child (29).

### 2.3. Statistical analyses

Before further analysis data was checked for skewness, kurtosis, outliers and normality. The Shapiro-wilk test was used to assess the normality of data distribution. Package “lessR” was used to calculate the means of the variables and differences between them (i.e., independent samples *t*-test) (30). Linear mixed effects models were used to estimate and compare activity duration, moderate-to-vigorous activity duration (MVPA in the equation), cycling duration and METs during cycling trips as compared to walking and car trips. The interaction between travel modes according to their duration (factor) was used as the fixed variable and participant (accounting for several trips from each participant) was used as the random variable as below:

$$\begin{array}{rl} Activity\;model=\; & lmer(Activity∼Travel\;mode\;\times \;Duration\\ & +(1+Travel\;mode\;\times \;Duration|Participant)) \end{array}$$

$$\begin{array}{rl} MVPA\;model=\; & lmer(MVPA∼Travel\;mode\;\times \;Duration\\ & +(1+Travel\;mode\;\times \;Duration|Participant)) \end{array}$$

$$\begin{array}{rl} Cycling\;model=\; & lmer(Cycling∼Travel\;mode\;\times \;Duration\\ & +(1+Travel\;mode\;\times \;Duration|Participant)) \end{array}$$

$$\begin{array}{rl} METs\;model=\; & lmer(METs∼Travel\;mode\;\times \;Duration\\ & +(1+Travel\;mode\;\times \;Duration|Participant)) \end{array}$$

Packages “lme4” and “lmerTest” were used to perform linear mixed model and to get *p*-values, respectively (31, 32). To test if the models are significant or not, we used Likelihood Ratio Test (31). For this mean we compared our models with a null model by replacing fixed value with gender. Package “table1” and “sjplot” were used to export results to table format (33, 34). Packages “ggplot2” and “ggeffects” were used to get the marginal means and confidence intervals for the models (35, 36). Finally, package “Epi” was used to calculate the sensitivity and specificity between Fibion-measured cycling during reported cycling trips versus walking and car trips (37). Because children's activities are sporadic (23), we tested different time windows (between >0 and >50 s) for the minimum duration of cycling required (38–40). Statistical analyzes were done using RStudio Version R-4.1.2 for windows. Statistical significance was set at *p* < 0.05 (two-tailed).

## 3. Results

Participants were a total of 44 boys and 116 girls aged 11.5 years (Table 1). They reported a total of 1,049 cycling trips (mean 6.6 trips per child), 379 walking trips (mean 2.5 trips per child) and 716 car trips (mean 4.5 trips per child) and accumulated a total of 2 h of cycling, 0.8 h of walking, and 1.5 h of car trips per week without differences between the genders (Table 1).

**Table 1:** Background and weekly trip characteristics by gender.

	All	Boys	Girls	*p*-value
	*N* = 160	*N* = 44	*N* = 116	
Age (years)	11.5 (0.88)	11.6 (0.91)	11.5 (0.87)	0.821
Height (cm)	153 (11.4)	153 (7.33)	154 (12.6)	0.695
Weight (kg)	47.3 (10.4)	47.5 (11.0)	47.2 (10.1)	0.878
Number of cycling trips per week	6.64 (4.80)	6.52 (4.29)	6.68 (4.99)	0.843
Cycling trip duration (h/week)	1.95 (1.42)	1.85 (1.15)	1.98 (1.52)	0.550
Cycling trip activity (h/week)	1.30 (1.08)	1.17 (0.89)	1.35 (1.14)	0.284
Cycling trip moderate-to-vigorous activity (h/week)	0.93 (0.81)	0.85 (0.65)	0.96 (0.86)	0.372
Number of walking trips per week	2.46 (2.85)	1.98 (2.26)	2.64 (3.03)	0.138
Walking trip duration (h/week)	0.79 (0.91)	0.67 (0.79)	0.84 (0.96)	0.239
Walking trip activity (h/week)	0.49 (0.64)	0.40 (0.56)	0.53 (0.67)	0.223
Walking trip moderate-to-vigorous activity (h/week)	0.40 (0.55)	0.31 (0.47)	0.43 (0.57)	0.154
Number of car trips per week	4.54 (4.04)	4.77 (3.69)	4.46 (4.18)	0.643
Car trip duration (h/week)	1.47 (1.33)	1.50 (1.18)	1.46 (1.39)	0.835
Car trip activity (h/week)	0.43 (0.45)	0.46 (0.40)	0.42 (0.46)	0.542
Car trip moderate-to-vigorous activity (h/week)	0.26 (0.30)	0.32 (0.30)	0.24 (0.30)	0.141

**Figure 1:**
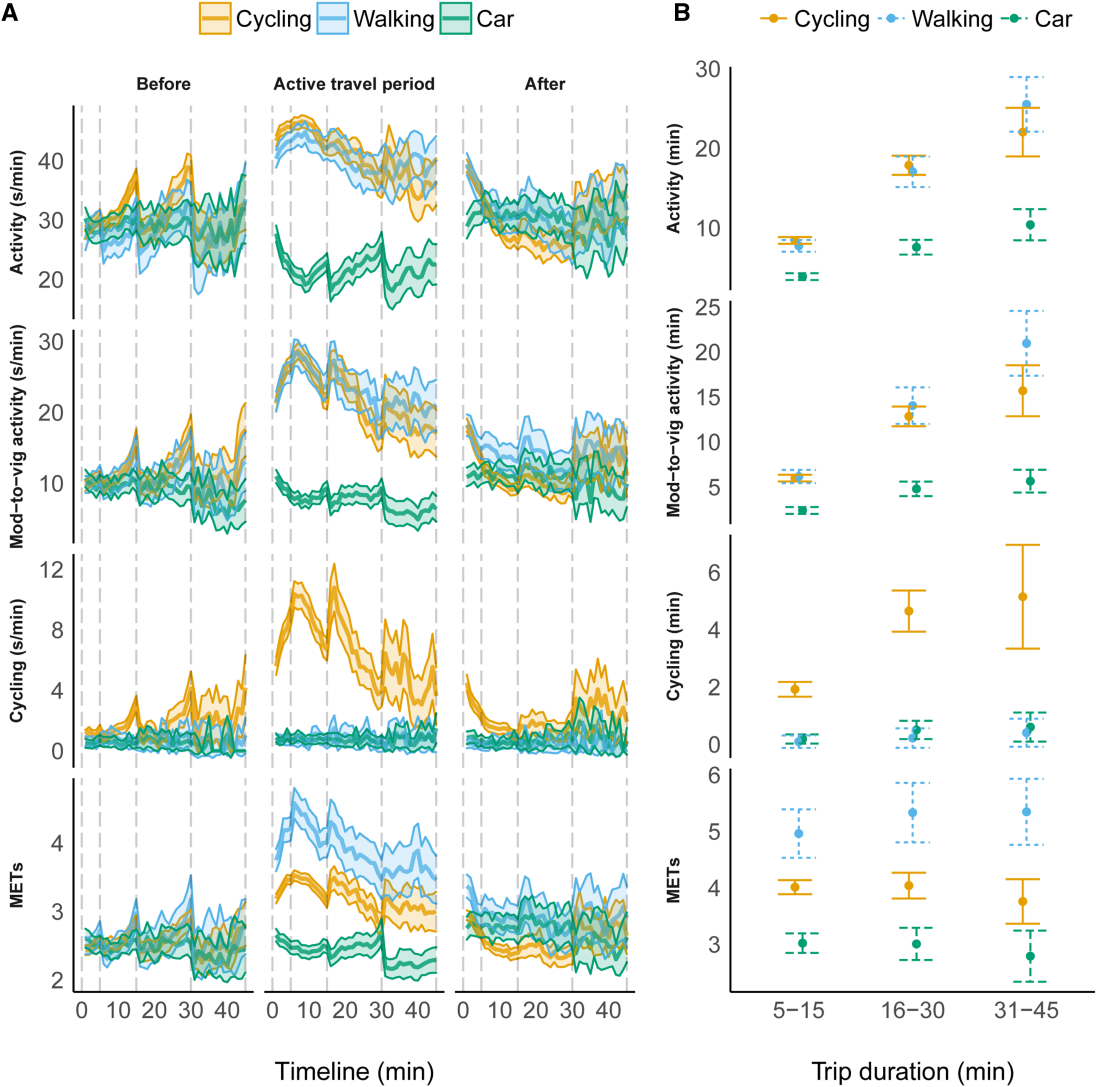
(**A**) Timeline of activity, moderate-to-vigorous activity, cycling, and METs before, during, and after the reported trip segments. The vertical dashed lines represent different travel log trip segment duration categories. (**B**) The estimated marginal means of activity, moderate-to-vigorous activity, cycling, and METs between travel modes during different trip segment durations.

Accumulation of accelerometer-measured physical activity before, during and after the segmented trips was visualized in Figure 1 to visually confirm that the reported trip segments increase physical activity. Figure 1 shows, that physical activity increases during cycling and walking trips, but decreases during car trips.

### 3.1. Linear mixed effects models

Linear mixed effects model results are presented in Table 2 and estimated marginal means in Figure 1B. There was no difference in activity and moderate-to-vigorous activity duration during walking trips as compared to cycling trips. As expected, there was a lower cycling duration during walking trips compared to cycling trips (−1.83 min, *p* < .001). Both activity (−4.54 min, *p *< .001), moderate-to-vigorous activity (−3.60 min, *p *< .001) and cycling duration (−1.74 min, *p* < .001) were lower during car trips as compared to cycling trips (Table 2).

**Table 2:** Activity, moderate-to-vigorous activity, cycling and METs estimated with linear mixed models using cycling as the reference trip.

Predictors	Activity (minutes)			Moderate-to-vigorous activity (minutes)			Cycling (minutes)			METs		
	Estimates	CI	*p*	Estimates	CI	*p*	Estimates	CI	*p*	Estimates	CI	*p*
(Intercept)	8.43	8.00 to 8.85	**<0.001**	5.98	5.59 to 6.36	**<0.001**	1.92	1.66 to 2.17	**<0.001**	4.01	3.89 to 4.14	**<0.001**
Car (vs. cycling)	−4.54	−5.14 to −3.94	**<0.001**	−3.60	−4.14 to −3.05	**<0.001**	−1.74	−2.04 to −1.43	**<0.001**	−0.99	−1.20 to −0.78	**<0.001**
Walking (vs. cycling)	−0.67	−1.48 to 0.14	0.105	0.18	−0.65 to 0.98	0.656	−1.83	−2.16 to −1.49	**<0.001**	0.95	0.53 to 1.37	**<0.001**
Trip duration 16–30 min (vs. 5–15 min)	9.44	8.32 to 10.56	**<0.001**	6.86	5.80 to 7.90	**<0.001**	2.73	2.10 to 3.37	**<0.001**	0.03	−0.22 to 0.27	0.826
Trip duration 31–45 min (vs. 5–15 min)	13.61	10.64 to 16.58	**<0.001**	9.73	6.96 to 12.52	**<0.001**	3.24	1.48 to 4.97	**<0.001**	−0.26	−0.66 to 0.15	0.213
16–30 min car trip (vs. 5–15 min cycling trip)	−5.74	−7.29 to −4.20	**<0.001**	−4.44	−5.84 to −3.05	**<0.001**	−2.42	−3.16 to −1.68	**<0.001**	−0.04	−0.42 to 0.34	0.840
16–30 min walking trip (vs. 5–15 min cycling trip)	−0.15	−2.28 to 1.99	0.893	1.07	−1.18 to 3.32	0.350	−2.61	−3.35 to −1.87	**<0.001**	0.34	−0.33 to 1.01	0.318
31–45 min car trip (vs. 5–15 min cycling trip)	−7.08	−10.23 to −3.93	**<0.001**	−6.50	−9.50 to −3.50	**<0.001**	−2.81	−4.63 to −0.98	**0.003**	0.03	−0.60 to 0.66	0.930
31–45 min walking trip (vs. 5–15 min cycling trip)	4.16	−0.21 to 8.54	0.062	5.28	0.88 to 9.67	**0.019**	−2.91	−4.74 to −1.09	**0.002**	0.64	−0.13 to 1.40	0.102

Bolded values indicate statistical significance at *p* < .05.

Trip segment duration reported in the travel log affected the measured activity duration, such that activity, moderate-to-vigorous activity, and cycling duration were longer during 16–30 min trips and 31–45 min trips as compared to 5–15 min trips (*p* < .001, Table 2).

Likelihood Ratio Test showed a significant change of activity (Chisq = 246.85; *p *< .001), moderate-to-vigorous activity (Chisq = 204.51; *p* < .001) and cycling (Chisq = 124.75; *p *< .001) between travel modes. In other words, Fibion® can detect a difference in these variables when these three travel modes are considered.

MET-level was significantly lower during car trips (−0.99, *p* < .001), but significantly higher during walking trips (0.95, *p* < .001) as compared to cycling trips (Table 2). Trip segment duration had no influence on the measured MET-level, suggesting that travelling intensity did not change as a function of trip duration. Likelihood Ratio Test indicated a significant change of METs between travel modes (Chisq = 126.60; *p *< 0.001), meaning that Fibion® measures a difference in METs between cycling, walking, and travelling by car (Table 2).

### 3.2. Sensitivity and specificity

Table 3 shows the results of sensitivity and specificity between Fibion® reported cycling and diary reported cycling versus walking and car trips. The required minimum duration of cycling had an influence on the sensitivity and specificity. When minimum required cycling duration was 29 s or less, sensitivity was 72.2% and specificity 81.9%. When the minimum duration of cycling was increased, sensitivity decreased (down to 63.0% with >50 s required for cycling) and specificity increased (up to 91.1% with >50 s required for cycling). Therefore, a higher minimum duration for cycling can be used to increase specificity, but for better sensitivity, the minimum duration required for cycling should be less than 29 s per trip.

**Table 3:** Sensitivity and specificity of Fibion-measured cycling during reported cycling trips (sensitivity) versus reported walking and car trips (specificity) using different minimum duration of Fibion-measured cycling.

Minimum required duration for cycling (s)	Sensitivity	Specificity	AUC
>0	72.4	81.8	0.77
>4	72.2	81.9	0.77
>9	72.2	81.9	0.77
>14	72.2	81.9	0.77
>19	72.2	81.9	0.77
>29	72.2	81.9	0.77
>34	69.7	83.8	0.77
>40	66.3	87.4	0.77
>50	63.0	91.1	0.77

AUC, area under the curve.

## 4. Discussion

The aim of this study was to investigate capability of a thigh-worn Fibion® accelerometer in estimating energy expenditure and physical activity during free-living cycling in 10–12-year-old children, which is a common challenge in device-based physical activity measurements. The main strength of this study is that we were able to analyze data from more than 2,144 free-living trips, including 1,049 cycling trips. The estimated differences between the travel modes showed that Fibion® captured more cycling, a lower MET-level, but a similar activity and moderate-to-vigorous activity duration during cycling trips as compared to walking trips. Because children's activity is often sporadic, the sensitivity and specificity were estimated by using different minimum duration criteria for measured cycling. The sensitivity and specificity were optimized when the minimum required duration for measured cycling was 29 s or less, but at a longer required cycling duration sensitivity decreased. This indicates the sporadic nature of children's free-living cycling and suggests that only a fraction of the cycling trip duration includes leg movement that the device recognizes as cycling. Despite previous studies have faced challenges in measuring physical activity during cycling using primarily waist-worn or wrist-worn devices, these results show that a thigh-worn accelerometer can be used to measure a similar activity and moderate-to-vigorous activity duration during free-living cycling trips as compared to walking trips.

### 4.1. Cycling has many health benefits, but physical activity during cycling is often underestimated

Free-living cycling is associated with a lower adiposity, a lower risk of cardiovascular diseases, a higher physical activity and a higher fitness in children and adolescents (4–8). Cycling is more beneficially associated with these outcomes as compared to other active travel modes, like walking (4–8). Yet, on many occasions physical activity during cycling has been significantly underestimated or rated as sedentary activity when using accelerometry (14, 15), even in controlled laboratory settings and compared to other activities such as walking (13). For this reason, several investigators have tried to find methods to measure cycling accurately and differentiate it from other activities.

In their validation study Herman Hansen et al. used a hip-worn ActiGraph in a sample of young adults during treadmill walking and ergometer cycling, and found no linear relationship between energy expenditure and activity counts during cycling. They reported that physical activity levels were underestimated by 73% during cycling when compared to walking (13). Similarly, Troutman et al. reported a low reliability for laboratory measured cycling (*R* = 0.05–0.75) as compared treadmill walking (*R* = 0.61–0.84) using an ankle and hip-worn Mini-Logger accelerometers in a sample of 10–16 years old children and adolescents (41). They also reported a weak validity for cycling (*r* = 0.06–0.15) and moderate validity for walking (*r* = 0.37–0.67) (41). Jakicic et al. found a significant association between energy expenditure of various activities (i.e., walking, running, stepping, and sideboard exercise) measured by a triaxial waist-worn accelerometer (TriTrac-R3D) and indirect calorimetry in young adults (10). However, they observed no association for cycling (10). Tarp et al. quantified the underestimation of free-living cycling activity measured with a hip-worn accelerometer (Actigraph GT3X) in 11–14 years old children (15). They noticed that cycling to and from school is an important source of physical activity for children and contribute substantially to moderate-to-vigorous physical activity levels, yet the accelerometer measured only 2.5%–3.3% of moderate-to-vigorous activity measured with heart-rate-monitor during cycling (15). The same result was also observed by Evenson et al. who used two hip worn accelerometers (ActiGraph and Actical) to estimate different activities in 5–8 years old children (14). Although both oxygen uptake (VO_2_) and heart rate were higher for stationary measured cycling than sedentary activities (e.g., watching a DVD, resting, and coloring) and walking they found that both accelerometers considered cycling as sitting/sedentary (14). Together these findings illustrate that the typically used wear locations and linear estimation equations result in underestimated cycling energy expenditure.

### 4.2. Thigh-worn accelerometers can measure cycling movement better as compared to wrist or waist worn devices

In contrast to the previous studies by Tarp et al. and Evenson et al., we observed a significantly higher moderate-to-vigorous activity duration during cycling trips than car trips, and no difference between walking and cycling. This means that Fibion® can capture cycling moderate-to-vigorous activity better than devices and analysis methods used in previous studies (14, 15). This may be due to that the movement during cycling can be better captured with a thigh-worn accelerometer as compared to hip, wrist or waist worn accelerometer, given the impacts in these locations are disproportionally associated with the energy expenditure of cycling. More generally, thigh worn accelerometers have been shown to differentiate activity types (e.g., sitting, standing, cycling, walking, and running) and intensity of activities better than hip or wrist worn accelerometers (19, 28, 42–45). Another difference to previously used methods is that Fibion® applies a different energy expenditure estimation algorithm for different activity types, whereas many of the previous studies have relied on linear associations between impacts and energy expenditure, with evident challenges in relation to cycling (14, 15).

Despite similar moderate-to-vigorous activity, the estimated MET-level was higher during walking trips compared to cycling trips. This is a similar result as in a recent study performed by Lucernoni et al., who included a sample of young adults and found that four ActivPAL (two ActivPAL3 & two ActivPAL4) attached on thigh underestimated METs by 33%–60% during stationary cycling (46). Yet, it should be noted that the present study was performed in free-living settings instead of in a laboratory-controlled setting. Lucernoni et al. concluded that ActivPAL does not provide accurate estimation of METs during cycling in a controlled lab setting (46). One possible reason for the underestimation of cycling METs in the present study may be the fact that capturing of cycling activity using Fibion® is based on continuous pedaling, whereas free-living cycling can also include interruptions, e.g., in traffic lights or during freewheeling. Walking does not include similar freewheeling periods that are possible during cycling. Thus, stops or not pedaling will be recorded as sitting or standing, but not cycling. Furthermore, using higher gears during cycling results in slower pedaling frequency despite the higher resistance, and the movement during cycling does not necessarily correspond to the actual effort or energy expenditure of cycling. These aspects of cycling are difficult or impossible to capture with accelerometers, and therefore physiological monitors should be used when investigators need to measure the physiological intensity of cycling.

There are a few studies using a single thigh worn accelerometer for detecting different outdoor activities in children and adolescents in free living conditions, and the existing studies have used short and controlled activity conditions (i.e., 1.5–5 min for each activity). Brønd et al. included 96 children and adolescents and used a single thigh worn Axivity AX3 accelerometer for detecting sitting, standing, walking, running, and biking (16). They used an activity log to record activity start and end times. They observed a high sensitivity and specificity (∼99%) for indoor measured activities such as sitting and standing but lower sensitivity and specificity (82.6%) for controlled, short, outdoor walking and running in both children and adolescents. They also reported sensitivity and specificity of ∼85.8% and ∼64.8% for identifying short controlled outdoor biking for children/adolescents and preschool children, respectively. They concluded that conducting a true free living validation study is challenging. In the present study we observed sensitivity of 72.2% and specificity of 81.9% (i.e., for time between 0 and 34 s), which is a similar magnitude to the study by Brønd et al., but included free-living, also longer duration, trips. Most recently, Bach et al. included 22 adults in their study and recorded their activities (e.g., sitting, standing, lying, walking, running, and cycling) during 1.5–2 h of free-living setting using direct video recording from chest, and dual accelerometers (Axivity AX3; worn on lower back and thigh) (47). Using machine learning methods, they observed that dual accelerometry can provide accurate estimation of free-living activities, but that a single thigh-worn accelerometer could also provide the same estimation. They reported sensitivity of 90% and specificity of 100% for the single thigh-worn accelerometer (47).

### 4.3. Sensor combinations can improve cycling intensity estimation but increase participant burden

One possible way for measuring free living physical activities (e.g., cycling) is using a combination of several devices at the same time, which allows for capturing not only activity types but also intensity, time, and distance of activities (48). For example, some studies suggested combination of both accelerometry, Global Positioning System (GPS) (49, 50) and heart rate (15) to measure cycling in children and adolescents. However, Brønd et al. argued that more complex algorithms or additional features (i.e., more accelerometers or other sensors) does not seem to help improving identification accuracy, but wear location and optimal selection of signal features may be more helpful (16). They suggested that more complex algorithms and increased amounts of features may increase the risk of overfitting leading to misclassification of some physical activity types in free living conditions. This applies especially to children whose activity behavior is sporadic and complex and thus difficult to capture (16). Thus, to achieve more accurate and better results, simple but robust methods such as single thigh-worn accelerometry should be considered.

In summary, while there are several recent studies using accelerometer/accelerometers combined with other sensors (e.g., heart rate, GPS) and analysing data with advanced statistical techniques, these high predictive values have been mainly provided models calibrated in laboratory conditions and are not necessarily reproducible in free-living conditions (51). This happens because of unseen and sporadic activities in free living conditions (43, 51, 52). Furthermore, it should be noted that using a combination of several sensors and statistical methods makes the process of measuring highly complex and difficult, in addition to the fact that this type of measurement is unfeasible in studies with large sample size and inconvenient for the participants (15, 52). Thus, it would be important to explore if a single sensor setup is capable in providing valid evaluations of key daily free-living activity types such as cycling (52). Such single-sensor measurements can be supplemented with, e.g., Ecological Momentary Assessment (EMA) (53), which is suggested to be more accurate than traditional self-report measures (54). Thigh-worn accelerometers are better in differentiating activity types and intensities compared to hip or wrist or waist-worn accelerometers (28, 45, 55). Although there are some studies showing excellent compliance with wrist-worn accelerometers in children (56), similar results have also been reported for thigh-worn accelerometers (17).

### 4.4. Strengths and limitations

The strength of this study is including a sample of children and their physical activity during several days in a free-living setting and using a simple method for estimation and evaluation of children's cycling behavior. The main limitation of the current study is using a travel log to capture the free-living cycling, walking and car trip segments. For example, the MET level during car trips was relatively high (∼3 METs). Rather than being an indication of MET level while sitting in car, the overall car trip segment also includes events immediately before and after the car trip, like walking to and from the car. We aimed to minimize this error by comparing different travel modes, which we assume are prone to similar error, and as such, focusing on estimated differences, rather than estimated marginal means, is recommended. Similar self-report measures have been used in some previous studies (16) and such a simple method enabled capturing a relatively high number of free-living trip segments. Moreover, Figure 1 shows that the reported trip segments included significantly more activity, moderate-to-vigorous activity, cycling, and a higher MET-level, as compared to segments recorded immediately before and after the reported trip segments. There was a higher number of girls than boys in the present study, which can be a real difference in their travel behavior but can also indicate under-reporting in boys. However, there were no differences in their overall weekly travel behavior suggesting that this bias, if any, should not affect the main results.

### 4.5. Conclusions

In conclusion, measuring physical activity during cycling has been difficult especially with hip, waist and wrist-worn accelerometers and due to relying on linear association between impacts and energy expenditure. Moreover, many of these studies have been conducted in controlled laboratory conditions, yet less data is available from free-living cycling. The present study quantified and compared activity, moderate-to-vigorous activity, cycling, and MET-level during more than 2,000 free-living trip segments in a sample of 10–12-year-old children. The thigh-worn Fibion® accelerometer measured a lower MET-level, but a similar activity and moderate-to-vigorous activity duration, and a higher cycling duration, during these reported cycling trips as compared to walking trips. Thigh-worn accelerometry can be used to measure free-living cycling activity and moderate-to-vigorous activity in children.

## Data Availability

The raw data supporting the conclusions of this article will be made available by the authors, without undue reservation.
