# Supplementary material for: Sensitivity and specificity of measuring children's free-living cycling with a thigh-worn Fibion® accelerometer
Source: Front Sports Act Living. 2023 May 23;5:1113687. doi: 10.3389/fspor.2023.1113687 (PMC10242071; doi:10.3389/fspor.2023.1113687)
Supplement: Supplementary file 1 [file Datasheet1.pdf]

FREERIDE –tutkimus **Tutkittavan tutkimusnumero**___________________

Ohjeet päiväkirjan täyttämiseen:
Selvitämme tällä päiväkirjalla arjen matkojen kulkutapoja yhden viikon ajalta. Lomakkeen ensimmäinen täyttöpäivä on sama kuin liikemittarin ensimmäinen mittauspäivä. Jatka päiväkirjan täyttämistä viikon ajan. Merkitse päiväkirjaan kunkin tutkimuspäivän **päivämäärä** sekä **heräämis- ja nukkumaanmenoajat.** Täytä päiväkirjalomakkeeseen **arjen matkat ruksilla** ja kerro tiedot yhdensuuntaisista matkoista –kaikki matkat merkitään erikseen
Irrota mittari **torstaina 3.6.** Palauta tämä päiväkirja ja liikemittari koululle **torstaina 3.6**

Tämä on esimerkki päiväkirjan täyttämisestä:

Tutkimuspäivän matkat

Esimerkkipäivän päivämäärä: 19.5.2021

Tutkimusviikon ensimmäinen päivä on kirjoitettu valmiiksi.

Moneltako heräsit tänään?
klo: 7.51
Moneltako menit nukkumaan tänään?
klo: 21.17

- **Piditkö liikemittaria koko päivän?**
  ☒ Kyllä
  ☐ Ei Mittari oli pois
  klo: _______ – klo: _________

- **Poikkesiko tämä päivä jotenkin tavanomaisesta?**
  Esim. Olit kipeänä
  ____

	Matka 1	Matka 2	Matka 3	Matka 4	Matka 5	Matka 6
**Mihin aikaan matka alkoi?**	8.15	14.05	15.00	15.45		
**Matkan tarkoitus**						
Koulumatka	☒	☐	☐	☐	☐	☐
Kotimatka	☐	☒	☐	☒	☐	☐
Ohjattu liikuntaharrastus	☐	☐	☐	☐	☐	☐
Muu ohjattu harrastus	☐	☐	☐	☐	☐	☐
Matka omatoimisesti liikkumaan (esim. skeittipuistoon)	☐	☐	☐	☐	☐	☐
Vierailumatka (kaverit ja muut tyypit)	☐	☐	☒	☐	☐	☐
Arkitoimet (esim. ostosmatka)	☐	☐	☐	☐	☐	☐
Muu vapaa-ajan matka	☐	☐	☐	☐	☐	☐
**Pääasiallinen kulkutapa**						
Kävellen	☐	☐	☒	☒	☐	☐
Pyörällä	☒	☒	☐	☐	☐	☐
Autolla	☐	☐	☐	☐	☐	☐
Bussilla tai koulukyydillä	☐	☐	☐	☐	☐	☐
Kävely tai pyöräily bussipysäkille	☐	☐	☐	☐	☐	☐
Skeitaten tai skootaten	☐	☐	☐	☐	☐	☐
Jokin muu	☐	☐	☐	☐	☐	☐
**Arvio matkan kestosta**						
alle 5 min	☐	☐	☒	☒	☐	☐
5–15min	☐	☐	☐	☐	☐	☐
16–30min	☒	☒	☐	☐	☐	
31–45min	☐	☐	☐	☐	☐	☐
yli 45 min	☐	☐	☐	☐	☐	☐

1. tutkimuspäivän matkat

1. tutkimuspäivän päivämäärä: ____________________________
Tutkimusviikon ensimmäinen päivä on kirjoitettu valmiiksi.

Moneltako heräsit tänään?
klo: ____________________________

Moneltako menit nukkumaan tänään?
klo: __________________________

- **Piditkö liikemittaria koko päivän?**

☐ Kyllä
☐ Ei **Mittaus alkoi klo:** ___________
Mittari oli pois
klo: ________ – klo: _________

- **Poikkesiko tämä päivä jotenkin tavanomaisesta?**

Esim. Olit kipeänä

____

	Matka 1	Matka 2	Matka 3	Matka 4	Matka 5	Matka 6	Matka 7	Matka 8	Matka 9	Matka 10
**Mihin aikaan matka alkoi?**										
**Matkan tarkoitus**										
Koulumatka	☐	☐	☐	☐	☐	☐	☐	☐	☐	☐
Kotimatka	☐	☐	☐	☐	☐	☐	☐	☐	☐	☐
Ohjattu liikuntaharrastus	☐	☐	☐	☐	☐	☐	☐	☐	☐	☐
Muu ohjattu harrastus	☐	☐	☐	☐	☐	☐	☐	☐	☐	☐
Matka omatoimisesti liikkumaan (esim. skeittipuistoon)	☐	☐	☐	☐	☐	☐	☐	☐	☐	☐
Vierailumatka (kaverit ja muut tyypit)	☐	☐	☐	☐	☐	☐	☐	☐	☐	☐
Arkitoimet (esim. ostosmatka)	☐	☐	☐	☐	☐	☐	☐	☐	☐	☐
Muu vapaa-ajan matka	☐	☐	☐	☐	☐	☐	☐	☐	☐	☐
**Pääasiallinen kulkutapa**										
Kävellen	☐	☐	☐	☐	☐	☐	☐	☐	☐	☐
Pyörällä	☐	☐	☐	☐	☐	☐	☐	☐	☐	☐
Autolla	☐	☐	☐	☐	☐	☐	☐	☐	☐	☐
Bussilla tai koulukyydillä	☐	☐	☐	☐	☐	☐	☐	☐	☐	☐
Kävely tai pyöräillä bussipysäkille	☐	☐	☐	☐	☐	☐	☐	☐	☐	☐
Skeitaten tai skootaten	☐	☐	☐	☐	☐	☐	☐	☐	☐	☐
Jokin muu	☐	☐	☐	☐	☐	☐	☐	☐	☐	☐
**Arvio matkan kestosta**										
alle 5 min	☐	☐	☐	☐	☐	☐	☐	☐	☐	☐
5–15min	☐	☐	☐	☐	☐	☐	☐	☐	☐	☐
16–30min	☐	☐	☐	☐	☐	☐	☐	☐	☐	☐
31–45min	☐	☐	☐	☐	☐	☐	☐	☐	☐	☐
yli 45 min	☐	☐	☐	☐	☐	☐	☐	☐	☐	☐
